# Coinfection with Different Feline Papillomaviruses in a Cat with Multicentric Squamous Cell Carcinoma In Situ

**DOI:** 10.3390/ani16152358

**Published:** 2026-08-02

**Authors:** Ana Rostaher, Nadine Angie Werlen, Paula Grest, Kurt Tobler, Anna Sophie Ramsauer

**Affiliations:** 1 Dermatology Unit, Clinic for Small Animal Internal Medicine, Vetsuisse Faculty, University of Zurich, 8057 Zurich, Switzerland; 2 Small Animal Hospital, School of Veterinary Medicine, University of Glasgow, Glasgow G12 8QQ, UK; 3 Institute of Virology, Vetsuisse Faculty, University of Zurich, 8057 Zurich, Switzerland; nadineangie.werlen@uzh.ch (N.A.W.); kurt.tobler@uzh.ch (K.T.); 4 Institute of Veterinary Pathology, Vetsuisse Faculty, University of Zurich, 8057 Zurich, Switzerland; grest@vetpath.uzh.ch; 5 Centre for Veterinary Public Health and One Health, Clinical Department for Farm Animals and Food System Transformation, University of Veterinary Medicine Vienna, 1210 Vienna, Austria

**Keywords:** carcinoma in situ, feline papillomavirus, imiquimod

## Abstract

A 14-year-old neutered male domestic shorthair cat was evaluated for long-standing, non-pruritic, crusty skin lesions on the pinnae, neck, and ventral abdomen that had persisted for at least 4 years. The histopathological examination of biopsies from the neck and abdomen revealed multicentric squamous cell carcinoma in situ confined to the epidermis. Papillomavirus diagnostics on ear crusts using PCR, rolling circle amplification (RCA), and next-generation sequencing (NGS) identified coinfection with Felis catus papillomavirus type 7 (FcPV7) and a putative novel feline papillomavirus type. This is the first reported case of multicentric carcinoma in situ with coinfection by these two papillomaviruses. The condition was successfully managed medically with topical imiquimod cream applied twice weekly for 3 months, resulting in complete clinical remission. Over 1.5 years of follow-up, they remained well. Two mild recurrences on the dorsal neck were effectively controlled with a short repeat course of imiquimod. This case report documents coinfection with different feline papillomavirus types in a cat and highlights an efficacious, practical, non-surgical treatment with the topical immunomodulatory imiquimod cream.

## Simple Summary

A 14-year-old cat was evaluated for long-standing, non-itchy, crusty skin lesions on the ears, neck, and abdomen that had recurred over several years. Cutaneous biopsies from the neck and abdomen showed multicentric squamous cell carcinoma in situ (an early form of skin cancer confined to the surface layer). Molecular testing of ear crusts using advanced DNA-based methods identified a previously unknown, putative novel feline papillomavirus type and Felis catus papillomavirus type 7 (FcaPV7), indicating co-infection. This represents the first report of multicentric squamous cell carcinoma in situ in a cat with coinfection of these two viruses. The cat was treated with a topical immune-modulating cream (imiquimod) applied twice weekly for three months, resulting in complete resolution of the skin lesions. During 1.5 years of follow-up, the cat remained well. Two small recurrences occurred and were rapidly controlled with short repeat courses of the same treatment.

## 1. Introduction

To date, there are ten fully sequenced Felis catus papillomavirus (FcaPV) types, within the *Papillomaviridae* family, that infect domestic cats according to the PapillomaVirus Episteme (PaVE; https://pave.niaid.nih.gov, accessed 3 March 2026). These FcaPVs are mainly associated with cutaneous hyperkeratotic plaques, oral and skin papillomas, multicentric squamous cell carcinoma in situ (MSSCISs), squamous cell carcinomas, basal cell carcinomas, Merkel cell carcinomas, and sarcoids [1,2,3].

Feline MSSCIS, also known as Bowenoid in situ carcinoma, is a rare premalignant skin disorder in cats and is considered analogous to Bowen’s disease in humans [4,5]. MSSCIS usually presents as multiple, irregular, scaly, and crusted papules or plaques with darker pigmentation at the periphery [5,6]. Furthermore, chronic lesions may ulcerate, and in rare cases, lesions become so keratinized that they form protruding keratin horns [5,6]. Predilection sites for MSSCIS include the head, neck, upper back, chest, and legs. Diagnosis of MSSCIS is established with biopsy and histopathology, with characteristic irregular skin and hair follicle thickening in areas with excessive keratin production and entire epidermal dysplasia [5,6]. Unlike ultraviolet-induced cutaneous squamous cell carcinoma, feline MSSCIS is generally not considered to be associated with chronic sun exposure [1].

Papillomaviruses (PVs) are host-specific epitheliotropic DNA viruses that infect skin and mucous membranes. Feline PVs were once called Felis domesticus PVs (FdPVs), but a more accurate name, Felis catus PVs (FcaPVs), has been suggested [7] and has since been widely adopted in the field; accordingly, we use the designation FcaPV throughout this manuscript. Currently, ten PVs specific for Felis catus have been described (https://pave.niaid.nih.gov, accessed 3 March 2026). The classified FcaPVs were assigned to the *Lambda*-, *Dyotheta*-, and *Taupapillomavirus* genera [8], and phylogenetic analysis suggested coevolution between these viruses and their hosts [9]. In general, PV infections are benign, result in a latent infection, or induce microlesions or benign neoplasias. However, a subset of human PVs, the so-called high-risk PVs (such as HPV16 and 18) and a few other animal PVs (such as Equus caballus PV2), are associated with the development of cancer [10,11]. Cancer progression is linked to persistent high-risk HPV infection and dysregulated viral gene expression, leading to excessive cell proliferation, impaired DNA repair, and the accumulation of genetic damage in infected cells [12]. This is considered to be related to the ability of E6 proteins to modulate p53 and PDZ-domain proteins, and to the capacity of E7 proteins to target multiple members of the retinoblastoma protein family [12].

While FcaPV-2 has been most commonly found in MSSCISs, other types of PVs, including FcaPV3, FcaPV4, and FcaPV5, have also been identified, but the exact role of the virus in the development of the disease has not been elucidated so far [1,13]. It was also shown that 98% of healthy cats carry FcaPV2. Still, only 22% mount an antibody response, suggesting that the virus is highly prevalent in this species but typically does not trigger an immune response [14].

The purpose of this study was to describe a case of coinfection with two papillomavirus types with MSSCIS in a cat with a relatively benign natural history that responded well to topical imiquimod treatment.

## 2. Case Presentation

A 14-year-old neutered male mixed-breed cat (body weight 4.3 kg) was presented with crusty skin lesions. These lesions were not pruritic and had been present for four years, with the crusts frequently detaching and then rapidly reforming. This cat was otherwise healthy, living indoors with another cat that does not exhibit any skin issues.

**Figure 1:**
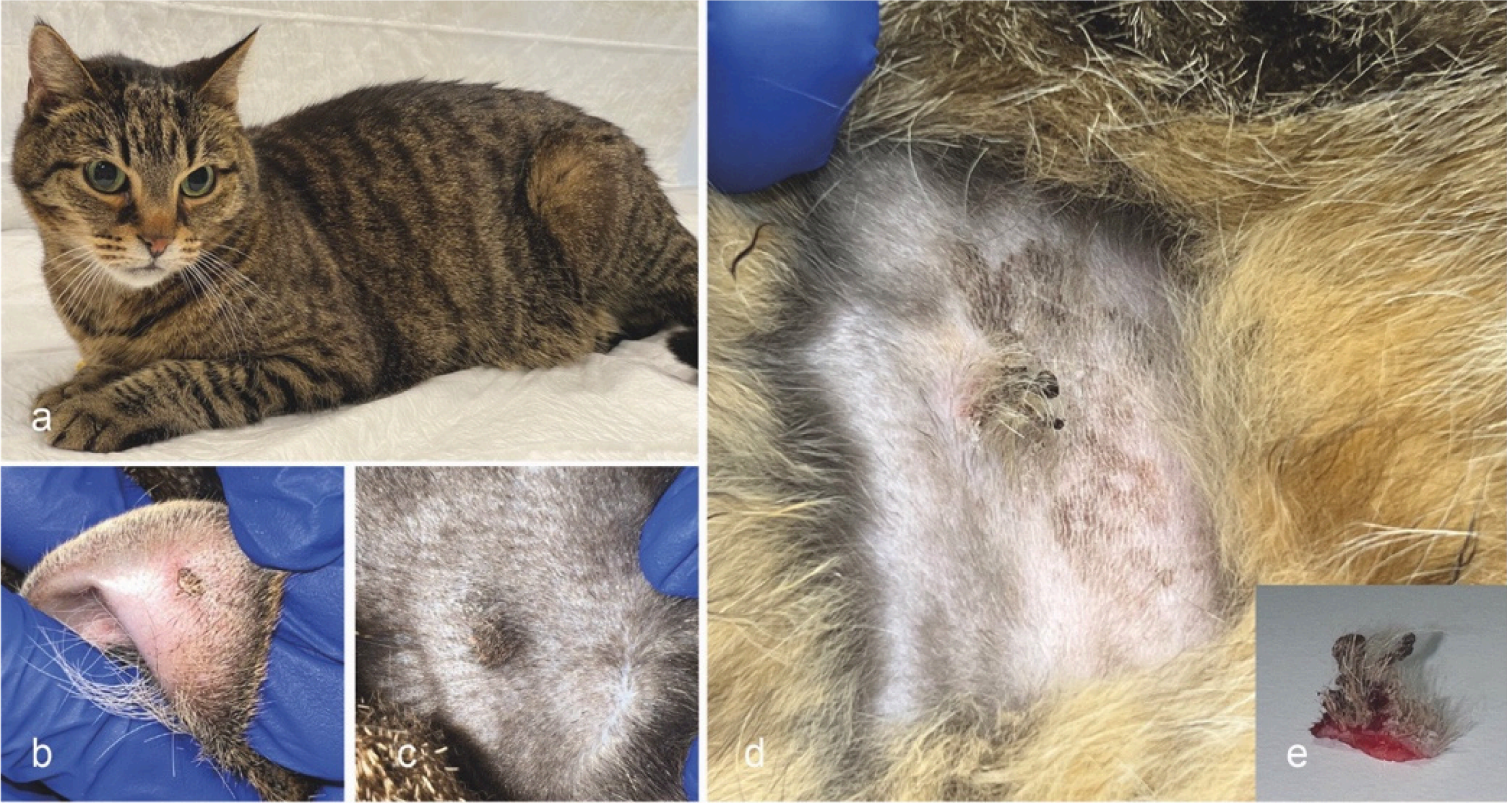
(**a**–**e**): The patient on the day of the biopsy (**a**). Multifocal crusts on the concave pinna (**b**), dorsal neck (**c**), and ventral abdomen (**d**) were observed. Additionally, the skin at the periphery of the lesion on the neck and abdomen was diffusely hyperpigmented (**d**). The insert (**e**) in (**d**) shows the removed skin tissue from the abdominal area after complete excision.

On physical examination, multiple crusty, partly scaly hyperpigmented plaques on the concave pinna, ventral abdomen and neck were observed (Figure 1a–e). The plaques on the pinnae and abdomen exhibited moderate erythema in the proximity (Figure 1b). The affected skin was moderately thickened, and lesion diameters ranged from 3 mm (ear) to 1 cm. After crust removal, the skin was eroded. The plaque on the abdomen showed two cutaneous horns in the central area and was surrounded by an approximately 2 × 8 cm large irregularly hyperpigmented area (Figure 1d). Based on signalment and clinical findings, the presumptive diagnosis was PV-associated (in situ) carcinoma. Other differential diagnoses included an inflammatory disease (e.g., bacterial infection), viral plaques, or another neoplastic mass.

Haematology revealed mild thrombocytopenia with a platelet count of 124 × 10^3^/µL (RI: 180–680 × 10^3^/µL); however, platelet clumping was seen, and the count was considered adequate. Elevated creatinine, urea, cholesterol, and glucose were detected in the serum biochemistry results. The creatinine was 221 µmol/L (RI: 98–163 µmol/L), the urea was 14.1 mmol/L (RI: 7.4–12.6 mmol/L), the cholesterol was 7.2 mmol/L (RI: 2.6–6.8 mmol/L), and the glucose was 19 mmol/L (RI: 4–9 mmol/L). The fructosamine was within the reference range (267 µmol/L; RI: 202–299 µmol/L). Serology testing for FIV and FeLV (Snap test, IDEXX) was negative.

Cytological examination of an impression smear taken beneath the crusts revealed mild neutrophilic inflammation with cocci and monomorphic epithelial cells, some of which were multinucleated. Additionally, a crust from the ear lesion was submitted for papillomavirus testing.

For papillomavirus detection and genotyping, total DNA was extracted from the ear crust using the QIAamp DNA Minikit according to the manufacturer’s protocol (Qiagen GmbH, Hilden, Germany). Initially, a conventional PCR was performed with primers targeting FcaPV2 (FcaPV2-E1-for: 5′-CAG CTC CCA GTC TCC TAA CG-3′; FcaPV2-E1-rev: GCT GTG CCA TTA TCT GAG CA-3′; and JMP [13]) and degenerate consensus (CP4/5 [15], FAP59/64 [16]) primers using REDTaq^®^ ReadyMix™ (Sigma Aldrich, Saint Louis, MO, USA) as previously described [10]. The ear crust tested positive for all primers (FcaPV2-E1, JMP, CP4/5, and FAP59/64). Positive bands of the consensus primers (CP4/5 and FAP59/64) were extracted from the gel, purified, and sent for bidirectional Sanger sequencing. The sequences were compared to other sequences on Genbank using BLASTN 2.17.0 (https://blast.ncbi.nlm.nih.gov/Blast.cgi, accessed 3 March 2026). The BLASTN analysis revealed only approximately 70% nucleotide identity to FcaPV-3 and FcaPV-6 across the aligned regions in both sequences, indicating the presence of a previously uncharacterized papillomavirus in the samples.

To further characterize the identified viral sequence, the circular viral DNA was amplified by Rolling Circle Amplification (RCA) using a TempliPhi amplification kit (GE Healthcare Life Sciences, Tokyo, Japan). The amplified DNA was then digested with the restriction endonucleases *Bam*HI and *Hind*III and purified using a Zymoclean Gel DNA Recovery Kit (Zymo Research, Irvine, CA, USA). Restriction enzyme analysis revealed a single *Bam*HI cut, yielding a fragment of approximately 7500 bp, and two *Hind*III fragments of approximately 5400 and 2100 bp. This pattern did not match the known FcaPVs.

Since Sanger sequencing using CP4/5 [15] and FAP59/64 [16], as well as RCA, suggested a putative novel PV, whole-genome (virome) sequencing was performed. The DNA was extracted from the ear sample and subjected to an established enrichment protocol for viral particles as previously described [17]. Then the nucleic acid samples were fragmented to 500 bp segments using Covaris E220 acoustic shearing technology. Library preparation was performed using Illumina-compatible NEBNext Ultra II DNA kits according to standard protocols (Illumina, San Diego, CA, USA). The prepared libraries underwent paired-end sequencing (2 × 150 bp format) on an Illumina NovaSeq 6000 instrument with an SP flow cell configuration (Illumina, San Diego, CA, USA). Quality control was ensured by incorporating Illumina’s PhiX v3 reference standard. After filtering, 26 million read pairs were available for further analysis. Subsequent computational analysis employed a previously established de novo assembly pipeline [18]. Two papillomaviral sequences could be assembled (designated as NODE #33 and NODE #39). 171 read pairs could be mapped to NODE #33 and 90,228 read pairs to NODE #39. The L1 CDS of these two genomic PV sequences were pairwise aligned to the corresponding feline PV references downloaded from PAVE, and the nucleotide identities were calculated (Table 1).

**Figure 2:**
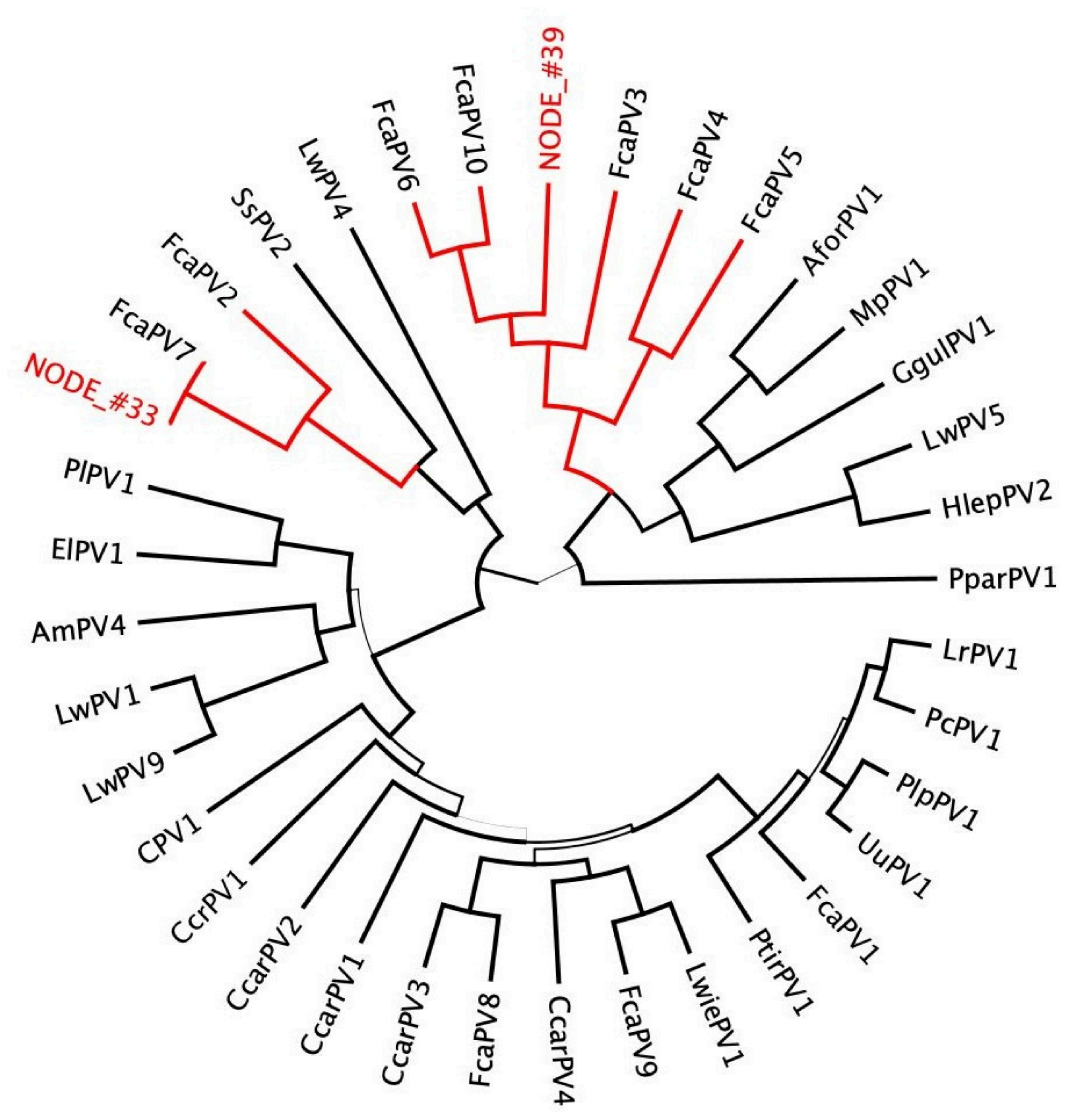
Bayesian phylogeny of L1 CDS. The line width corresponds to the posterior probability of the Bayesian model. The newly described PVs are marked in red. (Abbreviations: Arctocephalus forsteri papillomavirus 1 (AforPV1), Ailuropoda melanoleuca papillomavirus 4 (AmPV4), Canis familiaris oral papillomavirus 1 (CPV1), Caracal caracal papillomavirus 1 (CcarPV1), Caracal caracal papillomavirus 2 (CcarPV2), Caracal caracal papillomavirus 3 (CcarPV3), Caracal caracal papillomavirus 4 (CcarPV4), Crocuta crocuta papillomavirus 1 (CcrPV1), Canis latrans papillomavirus 1 (ClatPV1), Enhydra lutris papillomavirus 1 (ElPV1), Felis catus papillomavirus 10 (FcaPV10), Felis domesticus papillomavirus 1 (FcaPV1), Felis domesticus papillomavirus 2 (FcaPV2), Felis catus papillomavirus 3 (FcaPV3), Felis catus papillomavirus 4 (FcaPV4), Felis catus papillomavirus 5 (FcaPV5), Felis catus papillomavirus 7 (FcaPV7), Gulo gulo papillomavirus 1 (GgulPV1), Hydrurga leptonyx papillomavirus 2 (HlepPV2), Lynx rufus papillomavirus 1 (LrPV1), Leptonychotes weddellii papillomavirus 1 (LwPV1), Leptonychotes weddellii papillomavirus 4 (LwPV4), Leptonychotes weddellii papillomavirus 5 (LwPV5), Leptonychotes weddellii papillomavirus 9 (LwPV9), Leopardus wiedii papillomavirus 1 (LwiePV1), Mustela putorius papillomavirus 1 (MpPV1), Puma concolor papillomavirus 1 (PcPV1), Procyon lotor papillomavirus 1 (PlPV1), Panthera leo persica papillomavirus 1 (PlpPV1), Panthera pardus papillomavirus 1 (PparPV1), Panthera tigris tigris papillomavirus 1 (PtirPV1), Sus scrofa papillomavirus 2 (SsPV2), Uncia uncia papillomavirus 1 (UuPV1)).

Additionally, the phylogeny of the L1 coding sequences was visualized. Therefore, thirty-five L1 CDS reference sequences related to FcaPVs were collected from PaVE (https://pave.niaid.nih.gov). These reference sequences, together with the newly detected PV sequences, were aligned by MAFFT [20], and the Bayesian phylogeny was inferred with BEAUTi and BEAST [21]. The Bayesian search was performed with the GTRIG substitution model over 10 million generations, retaining the tree from every thousandth generation. The first 1000 trees in the set were burned in, and the remaining trees were summarized into a single target tree using TreeAnnotator [21]. The tree was edited with FigTree 1.4.4 [21] and is shown in Figure 2.

The two PVs detected as co-infection in the tissue samples are similar to FcaPV7 and FcaPV6/FcaPV10, respectively. NODE #33 is most likely a variant of FcaPV7 with 99.7% sequence identity and fairly identical to FcaPV2’s sequence. While NODE #39 is more distantly related, it is related to FcaPV6 and FcaPV10, sharing about 68% sequence identity, within a monophyletic clade that includes FcaPV3, 4, 5, 6, and 10. As the nucleotide identity of the L1 CDS at NODE #39 was below 70%, the newly detected PV genome is a putative novel PV type. The sequences were submitted to GenBank with the following accession numbers: NODE #33: PZ562381 and NODE #39: PZ562382.

Re-analysis of FAP59/64- and CP-4/5 amplicon-derived Sanger sequences and re-evaluation of RCA restriction patterns identified Node #39 in the original ear crust samples. Subsequently, specific PCR primers targeting NODE #33 and NODE #39 (Node-33-L1-for: 5′-GCA CCC TTA TTA CGA GCT GC-3′; Node-33-L1-rev: 5′-TGT CAA CCA TGT CAC CGT CT-3′; Node-39-L1-for: 5′-GGC GAA GGT TAT GAG CAC AG-3′; Node-39-L1-rev: 5′-CAC CTC GTC AGA TGC ACA TG-3′) were designed for conventional PCR analysis of lesions obtained during follow-up examinations.

**Figure 3:**
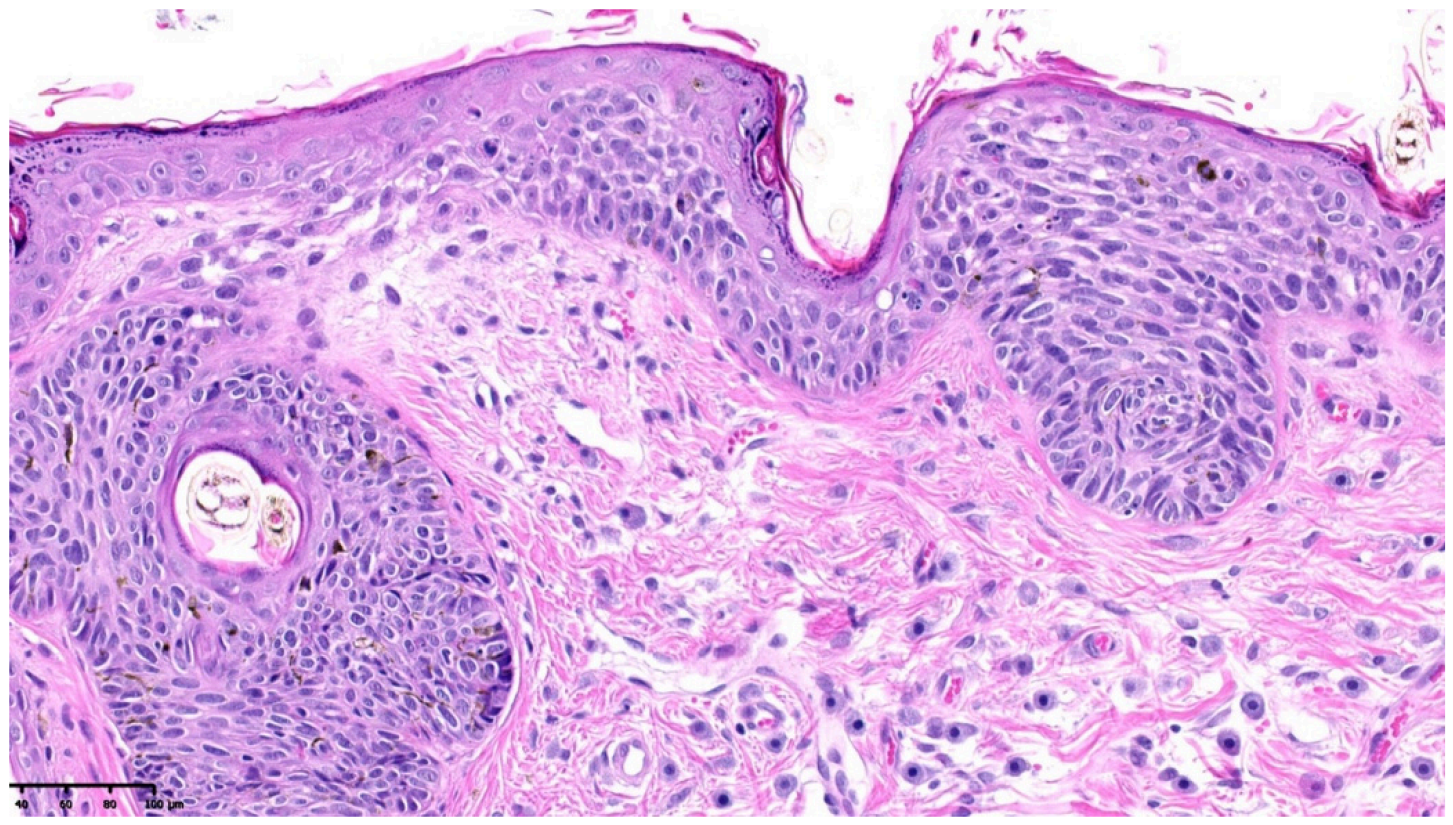
Skin, neck: Epidermal and infundibular hyperplasia with marked transepidermal dysplasia, characterized by a loss of nuclear polarity and disruption of normal epithelial stratification. Keratinocytes appear basaloid or display elongated nuclei, with moderate anisokaryosis. There is an increased number of intraepidermal melanocytes and pigmented keratinocytes.

At a follow-up examination one month later, photographic documentation of the potential PV-associated lesions (Figure 1) was obtained, and excisional biopsies of the skin lesions in the inguinal and neck areas and a shave biopsy from the ear were taken for histopathological examination and repeated papillomavirus analyses. Histopathological examination of the biopsied skin from the neck (Figure 3) and ventral abdomen revealed a well-demarcated area of moderate epidermal and infundibular hyperplasia and dysplasia without invasion through the basement membrane, with an abrupt transition between the lesional and non-lesional epidermis. These regions showed multifocal erosions, ulcerations, and crust formation. Dysplastic changes involved either the basal layer or the full thickness of the epidermis and infundibular epithelium and were characterized by loss of normal epidermal stratification and nuclear polarity. Keratinocytes displayed moderate pleomorphism and moderate anisocytosis with a predominantly basaloid appearance and scattered to multifocal groups of cells with elongated nuclei. Scattered mitotic figures were present in the upper layers of the epidermis, along with occasional apoptotic keratinocytes distributed throughout the epidermis. No koilocytic changes were observed. There was also an increase in pigmented cells. The superficial dermis showed a moderate mixed-cell infiltrate. The findings were consistent with a diagnosis of MSSCIS. The shave biopsies from the pinna revealed solely crusts consisting of keratin mixed with cellular debris, proteinaceous fluid, neutrophils, and coccoid bacteria. No epidermal or dermal tissue was present.

Subsequently, the crust from the ear and the two biopsies from the neck and inguinal region were resubmitted for papillomavirus testing by PCR using CP4/5, FAP59/64, FcaPV2-E1, Node-33-L1 and Node-39-L1 primers. All lesions tested positive with primers targeting FcaPV2 and FcaPV7/NODE #33, but were negative with primers specifically targeting NODE #39 and the degenerated consensus primers CP/4 and FAP59/64. It remains unclear if the primers targeting FcaPV2 detected FcaPV2 specifically or cross-reacted with FcaPV7/NODE #33 due to sequence similarities. However, no nucleic acids from the putative novel feline papillomavirus NODE #39 were detected in the follow-up samples anymore.

Based on the signalment and clinical findings, the presumptive diagnosis was PV-associated in situ carcinoma. The superficial bacterial infection was present as a secondary consequence of the primary disease (MSSCIS).

**Figure 4:**
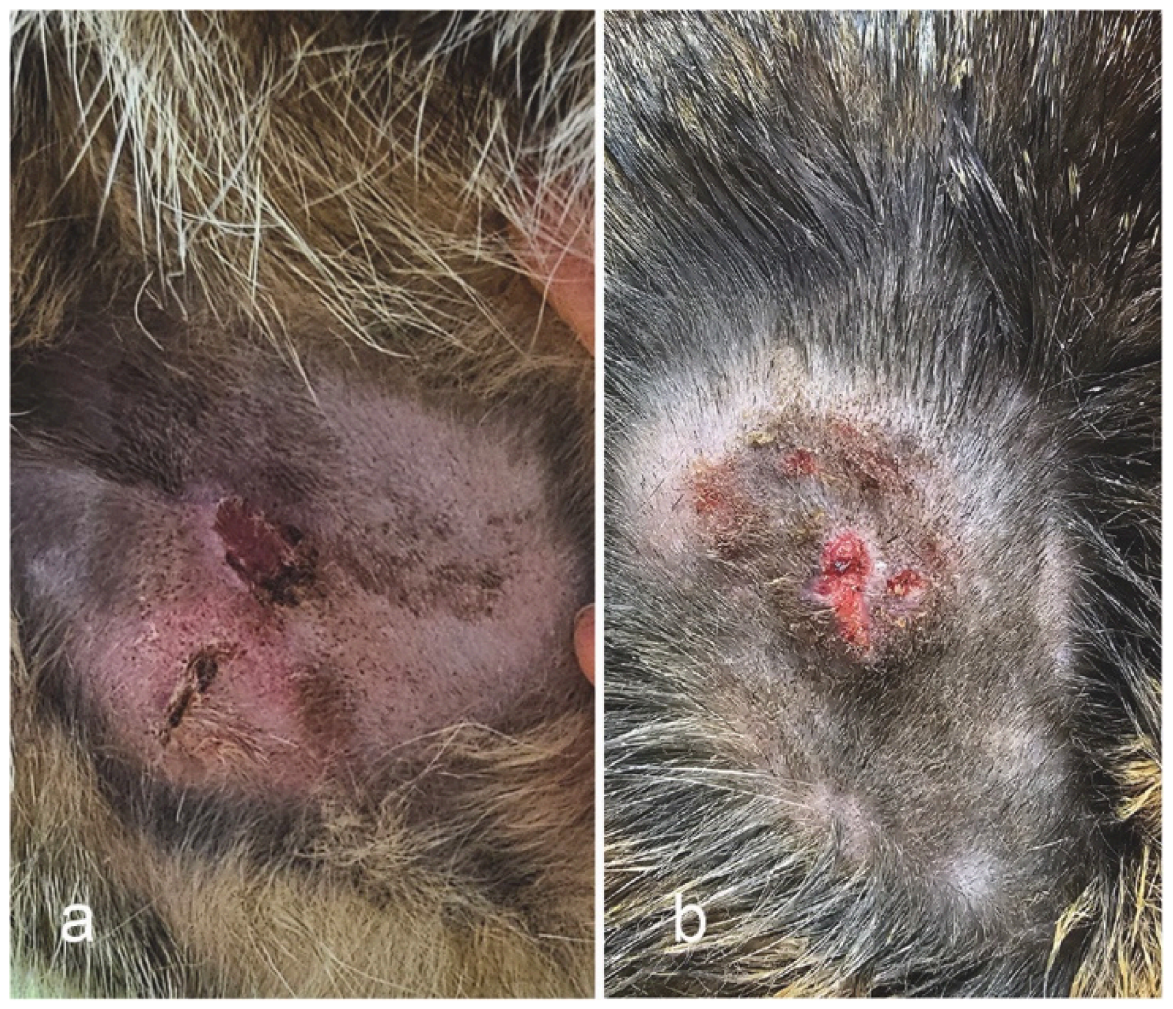
(**a**,**b**): A few days after the skin biopsy, the biopsied skin on the neck and inguinal area showed marked inflammation and relapse of crusting and scaling.

Ten days after the excisional biopsy, the biopsied skin area showed marked erythema, and the crusty lesions recurred (Figure 4a,b), indicating that surgical excision alone had not resulted in sustained remission. Consequently, topical imiquimod therapy was initiated to treat both the recurrent lesions and the remaining pinnal lesion.

Therefore, the cat was started on topical imiquimod cream (Aldara 5%, Viatris Pharma, Steinhausen, Switzerland) twice weekly. Following the manufacturer’s instructions, imiquimod 5% cream was applied as a thin layer to the affected lesions and a 1-cm margin of adjacent clinically normal skin. Owners were instructed to apply the cream in the evening and to use an Elizabethan collar to prevent grooming and accidental ingestion. The cream was left in contact with the skin overnight (approximately 8 h), after which any residual product was removed using a pad moistened with water. Treatment was well tolerated, and no local or systemic adverse effects were observed during the treatment period. The lesions slowly regressed over the next few weeks.

**Figure 5:**
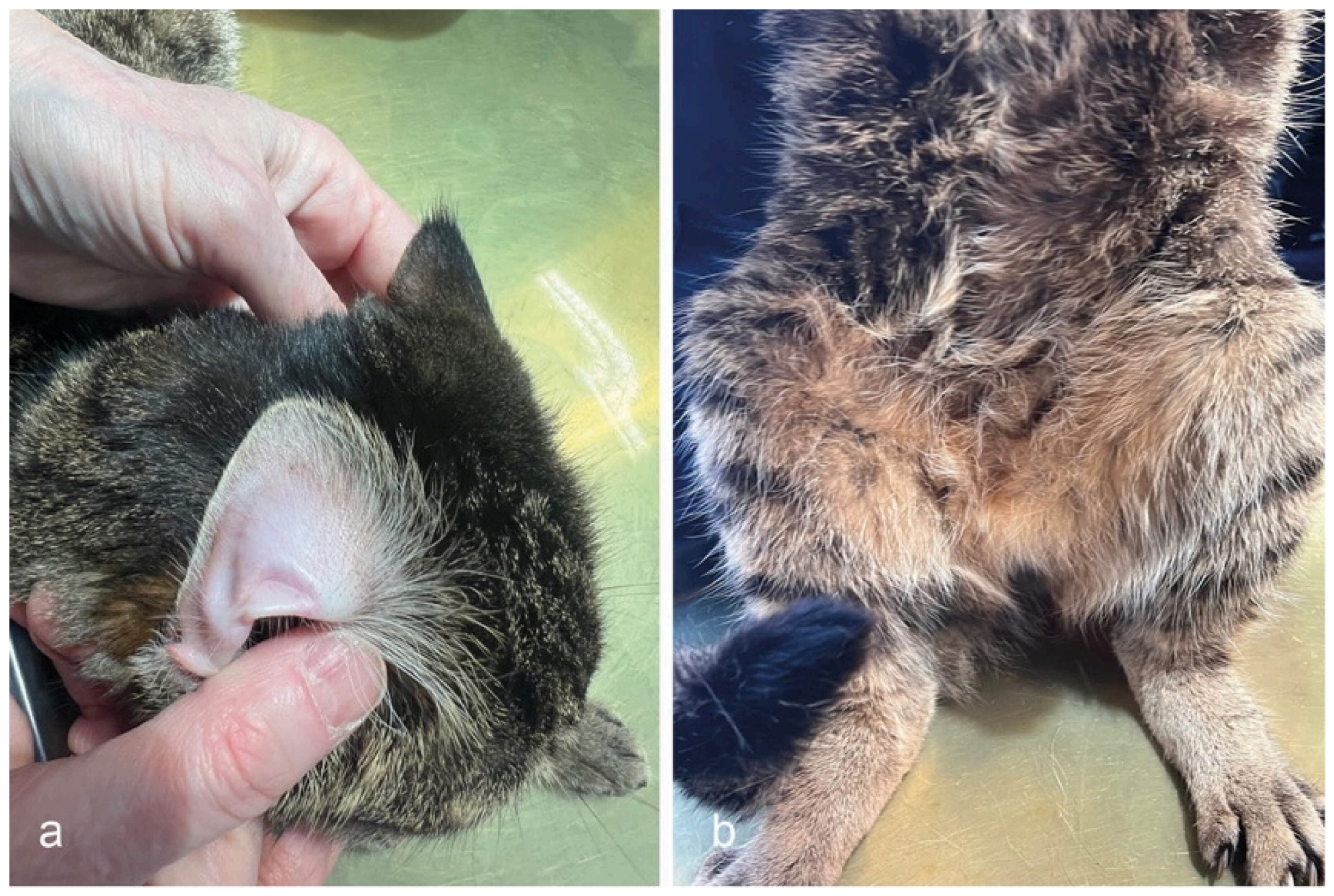
(**a**,**b**): At a follow-up recheck 1.5 years after the initial visit, the patient was in clinical remission. The skin on the pinnae (**a**) and inguinal area (**b**) was unremarkable.

The cat was in complete remission at the follow-up examinations 3, 6, 10, and 18 months after the initial visit. The owner reported two mild relapses in the neck area at months 7 and 11. These were milder than the initial episodes and responded well to a 2-week course of biweekly topical imiquimod application. The only observed side effect of imiquimod treatment was moderate exfoliation, which occurred exclusively at the neck application site and not in other treated regions. This reaction resolved promptly once the treatment was discontinued. Routine bloodwork performed at 1 and 10 months after initiating therapy showed no clinically significant deviations from baseline values, indicating that the medication was well tolerated systemically. At the 10-month follow-up examination, the cat exhibited a periocular nodule, which was fully excised. Histopathological examination of the periocular nodule revealed an apocrine ductular adenoma, and the papillomavirus PCR was negative. At the last follow-up recheck 1.5 years after the initial visit, the skin on the pinnae and inguinal area was unremarkable (Figure 5).

## 3. Discussion

This is the first report of an MSSCIS in a cat coinfected with these two PV types, expanding the known diversity of FcaPVs. Papillomaviral DNA has frequently been detected in feline MSSCIS lesions, but until now these sequences predominantly corresponded to FcaPV2. Lange et al. described the discovery of the newly described FcaPV2 in feline MSSCIS lesions [22], and Schwittlick et al. reported MSSCIS in association with FcaPV2 [23]. Additionally, other previously characterized PVs (such as FcaPV 1, 3, 4, 5) were detected in association with MSSCIS [24]. In contrast, the present case uniquely demonstrates concomitant infection with FcaPV7 and a putative novel PV type, for both of which no well-defined clinical presentation has been described to date. The novelty of this finding aligns with earlier observations that MSSCIS can be associated with papillomaviruses that differ genetically from established types and supports the concept that feline papillomavirus diversity is greater than currently recognized and that more PVs may play a role in MSSCIS development.

The co-infection with two different PV types is another rarely described, unusual, and noteworthy aspect in cats. Co-infection has not been well described in cats. However, the high background prevalence of PV DNA on feline skin makes the possibility biologically plausible, and it is a common event in human papillomavirus-associated cervical cancer [25]. Whether such co-infections modify disease expression, persistence, or immune evasion, thereby affecting disease progression and prognosis, remains unknown. However, coinfection by multiple PV types or variants could potentially increase oncoprotein burden, diversify targeted pathways, and enhance persistence, thereby potentially influencing lesion grade and clinical behavior. This case illustrates the need for broader genomic surveillance of feline PV infections to assess how frequently mixed infections occur and whether they influence clinical outcomes.

Surprisingly, in the follow-up PCR, we were unable to detect the putative novel FcaPV (NODE#39) again in the ear crusts and biopsy samples, which had initially exhibited a much higher read count in the NGS data. It remains unclear whether the cat cleared the infection with this virus within this short time frame, or whether other factors, such as differences in sampling sites, analytical sensitivity, or contamination, might have played a role. However, initial contamination appears unlikely, as this virus was detectable by three different methods (PCR, RCA, and NGS) in the initial sample, and we would generally expect higher analytical sensitivity with specific primers than with NGS. However, the sampling site and method may affect the results. The initial sampling affected only the ear crust, whereas the second sampling included biopsies of the neck and inguinal area. Potentially, the other lesions were never positive for the putative novel virus, or it was not detectable or cleared. While the first ear crust was clearly positive at the outset, the second, taken one month later, appeared negative. The second ear crust did not contain epidermal tissue and consisted of keratin mixed with cellular debris, proteinaceous fluid, neutrophils, and coccoid bacteria. Potentially, the virus was therefore not detected within these samples due to differences in the composition of the skin crust sample compared to the initial one.

An important consideration in the interpretation of this case is the lack of histopathological confirmation of MSSCIS in the pinna lesion. Although the lesion was clinically similar to the histologically confirmed lesions on the neck and ventral abdomen, yielded positive papillomavirus results, and responded similarly to treatment, the biopsy specimen consisted only of superficial crust material and lacked viable epidermal and dermal tissue for evaluation. The lesion was located in the central portion of the pinna, and a deeper biopsy was not pursued because of the risk of damaging the auricular cartilage and causing permanent structural and cosmetic defects. Therefore, although the pinna lesion was most likely part of the same disease process, its classification as MSSCIS remains presumptive rather than histologically confirmed.

As we detected the new virus solely in the ear lesion, we cannot show that there is an association with the coinfection with this virus and MSSCIS. However, FcaPV7 (NODE #33) was detectable in all follow-up samples, indicating a potential association with the presence of these lesions. To date, no clinical association has been described for FcaPV7.

Additionally, positive bands for primers targeting FcaPV2 were present in all samples. The positive FcaPV2 PCR result may have reflected a signal due to sequence similarity between FcaPV7 and FcaPV2. Still, coinfection with this third virus cannot be excluded, which would explain this clinical picture, as this association is well described [1,13] and as FcaPV2 is frequently present even in healthy cats [14]. However, as FcaPV2 was not detected by next-generation sequencing (NGS), we rather assume that only the two described viruses, and not FcaPV2, were present in the samples. Whether infection with a single virus among these viruses, and if so, which one, or coinfection with multiple viruses was responsible for the clinical disease remains questionable and warrants further investigation.

Another limitation of this report is the absence of in situ detection of papillomaviral nucleic acids or proteins within the lesions. At present, validated IHC antibodies and ISH probes for FcaPV7 and the putative novel papillomavirus are not available, and developing and validating bespoke assays was beyond the scope of this case report. Accordingly, we interpret our findings cautiously and refer to a possible/putative association rather than a proven causal link, particularly given that feline papillomaviruses can be detected in clinically normal skin. Nevertheless, detection of viral nucleic acids by multiple methods (PCR, RCA and NGS) and/or repeated PCR across multiple, time-separated specimens, together with the sequence data, supports the presence of these viruses in the affected areas.

In addition to the dermatological findings, routine laboratory investigations identified mild azotemia, hyperglycemia, and hypercholesterolemia. While it is unclear whether these findings were related to the development or persistence of the lesions, the presence of concurrent systemic disease may have influenced the host immune response. However, the significance of these abnormalities in the pathogenesis of MSSCIS remains speculative.

A notable aspect of this case is the overall mild clinical phenotype and the absence of metastasis or progression to invasive squamous cell carcinoma over a 1.5-year follow-up. This contrasts with some previously documented cases of MSSCIS, where progression to invasive SCC occurred in 17–25% of cats [23]. Although papillomavirus-associated SCCs in cats can behave aggressively, certain PV-associated SCC variants—such as the papillary SCC described by Munday et al. [26]—appear to have a more favorable prognosis. The benign course in the present cat, despite multifocal MSSCIS and long-standing lesions, is therefore noteworthy and may suggest distinct biological behavior potentially associated with these newly discovered PV types [23,26]. The phenotype in this case also differed from the more hyperkeratotic, often pigmented plaques commonly described in MSSCIS [1]. The lesions remained largely superficial, non-ulcerative, and clinically stable over several years before treatment initiation. Histopathology confirmed the absence of koilocytosis, a finding consistent with some earlier reports in which PV-induced cytopathic changes were inconsistently present. The absence of deep dermal invasion further supports the impression of a low-grade biological behavior.

Although surgery is traditionally recommended for MSSCIS [23], the present case demonstrates an excellent clinical response to topical imiquimod, further highlighting the suitability of immune-modulating therapy for potential papillomavirus-associated MSSCIS, as previously shown in a case series [27]. Because the neck and ventral abdominal lesions were excised before initiation of medical treatment, the therapeutic contribution of surgery cannot be completely separated from that of imiquimod. However, recurrence at the excision sites and subsequent remission following imiquimod therapy, together with resolution of the non-excised pinnal lesion, support a beneficial effect of topical imiquimod. Imiquimod functions as a Toll-like receptor 7 (TLR7) agonist, stimulating the local production of interferons and pro-inflammatory cytokines, thereby enhancing antiviral immunity and promoting clearance of papillomavirus-infected keratinocytes [28]. The favorable long-term control observed here underscores the advantages of a non-invasive, repeatable, immune-directed therapy—particularly in multifocal or chronic lesions where surgery may be impractical or cosmetically undesirable.

Recently, the use of CO_2_ laser ablation has also been reported as an emerging treatment option for papillomavirus-associated lesions [29]; however, this modality was not yet available at the time this case was managed and therefore could not be considered a therapeutic alternative.

## 4. Conclusions

This case provides new insights into the diversity, biology, and clinical behavior of feline potentially papillomavirus-associated skin disease. The discovery of a putative novel papillomavirus, the coinfection with different PV types, coupled with the benign clinical phenotype and favorable therapeutic response, highlights the need for further genomic and pathogenicity studies to clarify the role of emerging PV types in feline dermatopathology.

## Figures and Tables

**Table 1:** Percentage nucleotide identities of L1 coding sequences.

	NODE #33	NODE #39
NODE #33	100.0%	57.3%
NODE #39	57.5%	100.0%
FcaPV1	56.7%	57.5%
FcaPV2	72.1%	58.5%
FcaPV3	58.5%	69.9%
FcaPV4	58.1%	65.1%
FcaPV5	57.8%	62.5%
FcaPV6	58.2%	68.0%
FcaPV7	99.7%	57.6%
FcaPV8	55.9%	58.9%
FcaPV9	57.8%	56.0%
FcaPV10	56.2%	68.1%

Identities to FcaPVs calculated using ‘Needle’ from the ‘EMBOSS’ package 6.6.0.0 [19].

## Data Availability

All data necessary to replicate this analysis are contained within this article.
